# Mechanism of the blood-brain barrier modulation by cadherin peptides

**DOI:** 10.37349/eds.2024.00049

**Published:** 2024-06-26

**Authors:** Elinaz Farokhi, Ahmed L. Alaofi, Vivitri D. Prasasty, Filia Stephanie, Marlyn D. Laksitorini, Krzysztof Kuczera, Teruna J. Siahaan

**Affiliations:** 1 Department of Pharmaceutical Chemistry, School of Pharmacy, The University of Kansas, Lawrence, KS 66047, USA; 2 Current address: Analytical Department, Johnson & Johnson, San Diego, CA 92123, USA; 3 Current address: Department of Pharmaceutics, College of Pharmacy, King Saud University, Riyadh 11451, Saudi Arabia; 4 Current address: Department of Pathobiological Sciences, School of Veterinary Medicine, Louisiana State University, Baton Rouge, LA 70803, USA; 5 Current address: School of Pharmacy, University of Gadjah Mada, Yogyakarta 55281, Indonesia; 6 Department of Chemistry, The University of Kansas, Lawrence, KS 66047, USA; 7 Department of Molecular Biosciences, The University of Kansas, Lawrence, KS 66047, USA

**Keywords:** Ala-Asp-Thr peptides, blood-brain barrier, blood-brain barrier modulator, cadherin peptides, *trans*-cadherin interaction, NMR, molecular docking

## Abstract

**Aim:**

This study was aimed at finding the binding site on the human E-cadherin for Ala-Asp-Thr Cyclic 5 (ADTC5), ADTC7, and ADTC9 peptides as blood-brain barrier modulator (BBBM) for determining their mechanism of action in modulating the blood-brain barrier (BBB).

**Methods:**

ADTC7 and ADTC9 were derivatives of ADTC5 where the Val6 residue in ADTC5 was replaced by Glu6 and Tyr6 residues, respectively. The binding properties of ADTC5, ADTC7, and ADTC9 to the extracellular-1 (EC1) domain of E-cadherin were evaluated using chemical shift perturbation (CSP) method in the two dimensional (2D) ^1^H-^15^N-heteronuclear single quantum coherence (HSQC) nuclear magnetic resonance (NMR) spectroscopy. Molecular docking experiments were used to determine the binding sites of these peptides to the EC1 domain of E-cadherin.

**Results:**

This study indicates that ADTC5 has the highest binding affinity to the EC1 domain of E-cadherin compared to ADTC7 and ADTC9, suggesting the importance of the Val6 residue as shown in our previous in vitro study. All three peptides have a similar binding site at the hydrophobic binding pocket where the domain swapping occurs. ADTC5 has a higher overlapping binding site with ADTC7 than that of ADTC9. Binding of ADTC5 on the EC1 domain influences the conformation of the EC1 C-terminal tail.

**Conclusions:**

These peptides bind the domain swapping region of the EC1 domain to inhibit the *trans*-cadherin interaction that creates intercellular junction modulation to increase the BBB paracellular porosity.

## Introduction

Brain disorders remain a major challenge to treat because it is difficult to deliver therapeutic agents to the brain. This is because of the blood-brain barrier (BBB) that creates a physical barrier between brain parenchyma and blood circulation [1, 2]. The BBB is made of the blood microvessels with tight junction, adherens junction, and desmosome that seal the paracellular space [3, 4]. Various junctional proteins mediate cell-cell adhesion as zipper molecules at the intercellular junction that prevent diffusion of drugs with large hydrodynamic radius (> 11 Å) to permeate via the paracellular pathway [5]. Most of the small lipophilic drugs are transported via transcellular passive diffusion across the lipid bilayer; however, they can be subjected to efflux transporters that export them back to the lumen of BBB capillaries [1–3]. Medium and large molecules have difficulty penetrating the BBB via a passive diffusion mechanism; thus, these molecules normally enter the brain by specific transporters. Unfortunately, this route requires a specific molecular structure to be recognized by a certain transporter [1–3].

An alternative method to enhance the brain deposition of molecules with various sizes is by disrupting cadherin homophilic interactions at the adherens junction to create large pores in the BBB paracellular pathway [1]. Cadherin-cadherin interactions can occur via two different mechanisms. The first mechanism involves homophilic *trans*-interactions between two extracellular-1 (EC1) domains (EC1-to-EC1) of cadherins from opposite cells [6–8]. The second mechanism is via a *cis*-interaction in which two neighboring cadherin molecules from the same cell membranes interact using their respective EC1 and EC2 domains [6–8]. A domain swapping mechanism occurs in a *trans*-interaction of two cadherins from the cell membranes that are facing each other (opposite); in this case, the N-terminal of the EC1 domain of one cadherin docks to the EC1 hydrophobic pocket of another cadherin and vice versa [6].

The BBB paracellular porosity can be magnified by Ala-Asp-Thr (ADT) and His-Ala-Val (HAV) peptides that were derived from cadherin sequences to inhibit cadherin-cadherin interactions [9, 10]. The ADT and HAV peptides were designed from the amino acid sequences of the bulge and grove regions in the cadherin EC1 domain, respectively [9, 10]. As BBB modulators (BBBMs), both ADT and/or HAV peptides enhanced brain depositions of various sizes of molecules to the brain parenchyma such as IRdye 800CW, ^3^H-daunomycin, adenanthin, or gadolinium-diethylenetriaminepentacetate (Gd-DTPA or gadopentetic acid), peptides, 76 kDa albumin, and 150 kDa monoclonal antibody (mAb) [10–15]. Therefore, cadherin peptides or BBBMs can be used as an adjuvant to enhance the delivery of therapeutic or diagnostic agents into the brain for brain diseases such as Alzheimer’s disease (AD), brain tumor, and multiple sclerosis (MS). We hypothesized that the mechanism to modulate cadherin-cadherin interactions by ADT and HAV peptides is different. An HAV peptide, cyclic HAV-Cys 3 (cHAVc3) (Table 1), increased the porosity of the paracellular route through binding to the EC1 domain followed by disruption of the *cis*-cadherin interactions [16–20].

Currently, the mechanism of binding as well as the EC1 binding site of ADT peptides have not been elucidated; thus, this study was aimed at using nuclear magnetic resonance (NMR) and molecular docking experiments to determine ADTC5 binding properties (Table 1) on EC1. Our previous study suggested that the Val6 residue in ADTC5 has a big effect on its activity; thus, mutation of this residue reduced inhibitory activity in preventing the resealing of the paracellular pathway of Madin-Darby canine kidney (MDCK) cell monolayers [21]. Therefore, the Val6 residue was mutated to the Glu6 or Tyr6 residue in ADTC7 and ADTC9, respectively (Table 1). In this study, ^15^N-EC1 protein was utilized to probe the binding properties of these cyclic peptides. Peptide titration of the EC1 domain caused chemical shift perturbations (CSPs) of EC1 amino acids when detected using ^1^H-^15^N-heteronuclear single quantum coherence (HSQC) NMR spectroscopy. The peptide binding sites on EC1 were determined and visualized using NMR constraints on the docking simulation experiments. The ADTC5 binding characteristics on EC1 will be used to design derivatives with high affinity and specificity for the EC1 domain.

## Materials and methods

### Solid-phase peptide synthesis

ADTC5, ADTC7, or ADTC9 was synthesized using Fmoc chemistry and solid-phase peptide synthesis in an automated peptide synthesizer (Tribute, Gyros Technology, Tucson, AZ, USA) according to the previous study [21]. Each peptide was removed from the 4-methylbenzhydrylamine (MBHA) resin using a cocktail solution of trifluoro acetic acid (TFA)/anisole/ethanedithiol (EDT). Purification of the crude peptide was achieved utilizing C18 semi-preparative column (130 Å, 5 μm, 30 mm × 250 mm, XBridge, Water) in high performance liquid chromatography (HPLC) system (Rainin Dynamax, Varian). Peptide cyclization was accomplished using air oxidation to facilitate the formation of a disulfide bond between two cysteine residues. Cyclic peptide fraction was analyzed with C18 analytical column (Microsorb MV100-5, 100 Å, 4.6 mm × 220 mm, Agilent) in analytical HPLC system (Agilent 1100 Series HPLC) and pure fractions were combined and lyophilized. Pure peptide was characterized using electron spray ionization (ESI) mass spectrometry.

### Expression and purification of ^15^N-labeled EC1 domain

Our previous method was used to express and purify the ^15^N-labeled EC1 domain [22]. Briefly, the plasmid (pASK-IBA6, Genosys, Woodland, TX) contains DNA construct of 138 amino acid residues. The EC1 domain has 110 residues connected to 28 amino acids from the EC2 domain sequence at the C-terminus tail followed by a WSHPQFEK sequence of Streptag I that is linked by an IEGR sequence for a factor Xa cleavage. Then, the plasmid was transformed into competent *E. coli* BL21(DE3) cells (New England Biosciences, Ipswich, MA). The cells were incubated at 37°C overnight on a plate with lysogeny broth (LB) agar containing 100 mg/mL ampicillin. A single colony of *E. coli* cells was picked and incubated in 10 mL of LB media followed by the addition of 10 μL ampicillin at 100 mg/mL concentration. Then, the mixture was incubated in an orbital shaker at 250 rpm and 37°C. ^15^N-labeled NH_4_Cl was added to 250 mL of M9 minimal media in 1 L Erlenmeyer flask to produce the ^15^N-labeled EC1 domain. A 24 μL of anhydrotetracycline (2 mg/mL; Promega Inc., Madison, WI) was spiked into the cell culture when it reached OD550 of 0.5–0.6 to induce the EC1 protein expression. Then, the cell mixture was incubated at 30°C for 6–7 h followed by cell harvesting using centrifugation at 4,500 xg. The resulting cell pellets were stored at −80°C.

For isolation of purified ^15^N-EC1, *E. coli* cell pellets were lysed using 20,000 psi French press (Thermo Electron Corporation) in 10 mL of binding buffer. This binding buffer consists of 150 mM NaCl, 1.0 mM EDTA, 0.02% NaN_3_, and 100 mM Tris-HCl at pH = 8.0. The mixture was centrifuged at 21,000 xg, 4°C, and 1 h to get rid of cellular debris. The EC1 domain was in the supernatant and the Amicon Ultra protein concentrator [10 kDa molecular weight (MW) cut-off, EMD Millipore, Billerica, MA] was used to concentrate the protein upon centrifugation at 4,500 rpm, 20 min, and 4°C. The Strep-Tactin II affinity column (5.0 cm × 0.6 cm, GE Healthcare) was used to purify the EC1 domain at room temperature. The binding buffer was used to elute the unbound proteins from the column. Next, the pure EC1 domain was eluted from the column at a flowrate of 2 mL/min using a buffer containing 1.0 mM dithiothreitol (DTT) along with 2.5 mM desthiobiotin at pH = 8.0. Fractions containing EC1 were concentrated to 1.5 mL. The purity of pooled EC1 fractions was evaluated by SDS-PAGE. Protein concentration was calculated using UV spectrometry at 280 nm (coefficient absorption = 19,480 M^–1^ cm^–1^). The storage of pure ^15^N-labeled EC1 was done in an elution buffer at 4°C.

### Titration of the EC1 domain with ADT peptides in NMR

A total of 564 μL of 0.35 mM purified ^15^N-labeled EC1 in 20 mM Tris-HCl buffer was dialyzed overnight using Slide-a-Lyzer dialysis cassette (5.0 kDa MW cut-off, Thermo Scientific, PA) in 1 L NMR buffer containing 20.0 mM potassium phosphate and 5.0 mM DTT at 4°C and pH = 6.0. After dialysis, 36 μL of 6% D_2_O was added to the protein solution. Protein and peptide mixtures for NMR titration were prepared with 20 μM, 0.18 mM, and 0.4 mM of stock solutions for ADTC5, ADTC7, and ADTC9, respectively. The protein and peptide solutions were mixed according to the ratio used for the NMR study.

A 600 MHz Bruker Avance NMR spectrometer (Bruker Inc.) furnished with a triple resonance probe (Bruker, Inc.) was used to collect the ^1^H-^15^N HSQC data. The 2,048 data points were collected in ^1^H resonance with 16 scans and ^15^N resonance in 128 increments. The NMR solution was spiked with 4,4-dimethyl-4-silapetane-1-sulfuric acid (DSS) dissolved in D_2_O for the internal standard. An NMRpipe under the SPARKY program was used to process the NMR data and determine the shift changes in the ^1^H-^15^N HSQC spectra [23].

### Combination of NMR and molecular docking experiments

To prepare the EC1 domain virtually, a free EC1 three dimensional (3D) structure (PDB ID: 2O72) was put in a box and solvated with water molecules for molecular dynamics (MD) simulations. Counter ions were added to simulate the ionic state of EC1 at a neutral pH. The system was minimized and MD simulation was carried out for 100 ns at 300 K. The last frame from the MD trajectories was captured to be used in the docking simulation. The InsightII program (Accelrys, Inc., San Diego, CA) was utilized to build the 3D structures of ADTC5, ADTC7, and ADTC9 followed by structural minimization using CVFF91 forcefield. The structures were saved in pdb file format for the next step.

Dockings of ADT peptides on EC1 were accomplished using the HADDOCK program along with the Easy Interface option [24]. The “active” amino acids on EC1 were assigned using the data from NMR CSP caused by each peptide. Noticeable CSPs on EC1 when titrated with ADTC5, ADTC7, and ADTC9 peptide were designated as “active” residues. Docking simulations with HADDOCK program were carried out using active residues [25]. Clusters with the highest scores were selected for further analysis to generate working models for peptide-protein binding. Analysis of the docking cluster generated by HADDOCK was done using LigPlot program to view the 2D interaction diagram between the peptide and EC1 [26].

## Results

### ADT peptide binding properties on EC1 evaluated by ^1^H-^15^N HSQC NMR

**Figure 1.**
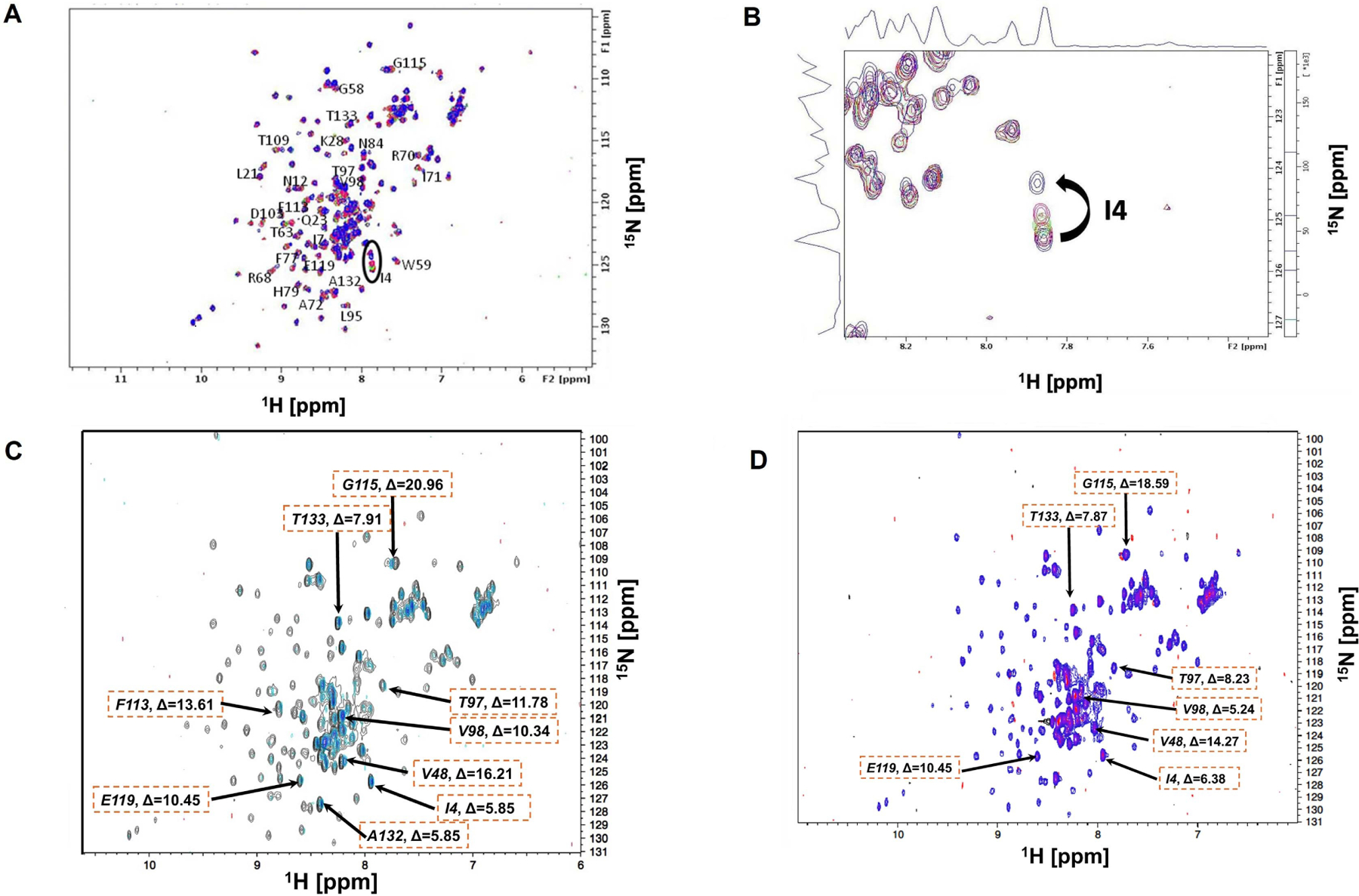
Changes in NH chemical shifts of the extracellular-1 (EC1) domain amino acids in two dimensional (2D) ^15^N-heteronuclear single quantum coherence (HSQC) spectra (600 MHz) upon titration with Ala-Asp-Thr (ADT) peptides. The protein was dissolved at 0.4 mM concentration in 20 mM KH_2_PO_4_ and 5.0 mM dithiothreitol (DTT) buffer at pH 6.0. (A) Overlayed spectra of EC1 alone (purple) and EC1 + ADT cyclic 5 (ADTC5) (red) at 1:3 protein-to-peptide ratio; (B) the magnification of I4 chemical shifts upon the addition of different concentrations of ADTC5; (C) overlayed spectra of EC1 alone (black) and EC1 + ADTC7 (blue) with 1:15 protein-to-peptide ratio. The EC1 domain at 0.18 mM concentration was dissolved in a buffer containing 5.0 mM DTT and 20 mM KH_2_PO_4_ at pH 6.0; (D) overlayed spectra of EC1 alone (purple) and EC1 + ADTC9 (pink) in 1:15 protein-to-peptide ratio. (C–D) The ΔF values for affected residues were shown as the maximum change

Each peptide was titrated into the labeled EC1 solution and CSPs of amino acids were observed upon titration (Figure 1). The spectra of the ^15^N-labeled EC1 domain in 2D-HSQC NMR were collected before and after titrations as in the previous study [16, 22]. Backbone assignments for most of the NH cross-peaks have been done from the previous study with the exception of several residues: E64, L66, V81, I96, Q101, E111, M128, L122, A135, and D136 [22]. Most of the conserved residues have been assigned for the CSP determination in the next step.

The peptides were added to the labeled EC1 solution with increasing ratio and the changes in spectra under the same physical condition were quantified as CSP. A combination of ^1^H and ^15^N chemical shifts was used as the overall shift (ΔF) after titration and the following equation was used to calculate ΔF [27]:

$$\Delta \text{F=}\left[{\left(\Delta \delta^{1}\text{H}*599.743\right)}^{2}+{\left(\Delta \delta^{15}\text{N}*60.778\right)}^{2}\right]$$

The EC1 domain interaction with ADTC5 peptide with ratios between 1:1 and 1:3.3 was investigated under the same physical condition as the EC1 domain alone. The overlayed NMR spectra for ADTC5 titration were visualized in Figure 1A. Some of the cross-peaks appeared to be shifted upon the addition of ADTC5 peptide. One of the dramatic changes was seen for residue I4 with larger CSP compared to other residues upon increasing peptide concentrations (Figure 1B).

**Figure 2.**
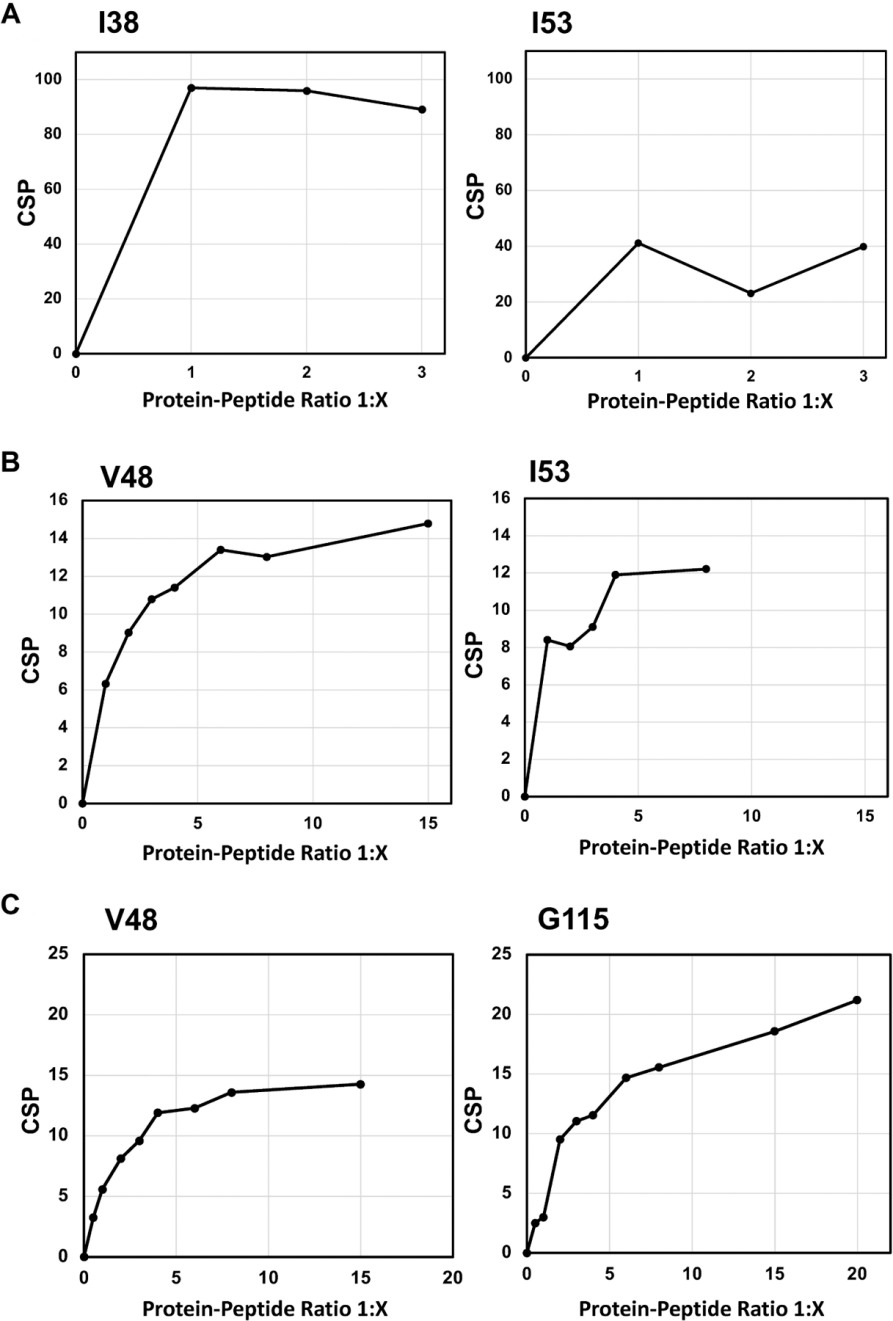
The effects of titration with various concentrations of (A) Ala-Asp-Thr Cyclic 5 (ADTC5), (B) ADTC7, and (C) ADTC9 peptides on the NH chemical shifts of active residues on the extracellular-1 (EC1) domain. (A) The effect of ADTC5 on overall chemical shift changes and saturations of the I38 and I53 residues; (B) the effect of ADTC7 on overall chemical shift changes and saturations on the V48 and I53 residues; (C) the effect of ADTC9 on overall chemical shift changes and saturations of the V48 and G115 residues. CSP: chemical shift perturbation

In the NMR study of ADTC5, the majority of the EC1 domain peaks shifted after titration with 1:2 protein-to-peptide ratio. The shift in the I4 residue upon peptide addition was observed at 1:1 protein-to-peptide ratio, and as the peptide concentration increased, the cross-peak was also dramatically shifted at 1:3 protein-to-peptide ratio (Figure 1B). In addition to the strong effect of the NH of I4 residue, significant shifts were also observed in the NHs of I38, I53, V98, D103, and F113 residues. The individual saturation curves for selected residues (I38 and I53) are shown in Figure 2A. These shifts could be due to direct binding or allosteric effects. The CSPs for most residues in the EC1 domain were plateaued at 1:3 to 1:3.3 protein-to-peptide ratios presumably due to ADTC5 binding saturation. Other residues that moderately affected by titration include I7, S8, N12, Q23, K28, Y36, G58, W59, T63, R68, I71, A72, F77, S78, H79, N84, L95, T97, V98, D103, T109, F113, G115, E119, A132, and T133 residues.

**Figure 3.**
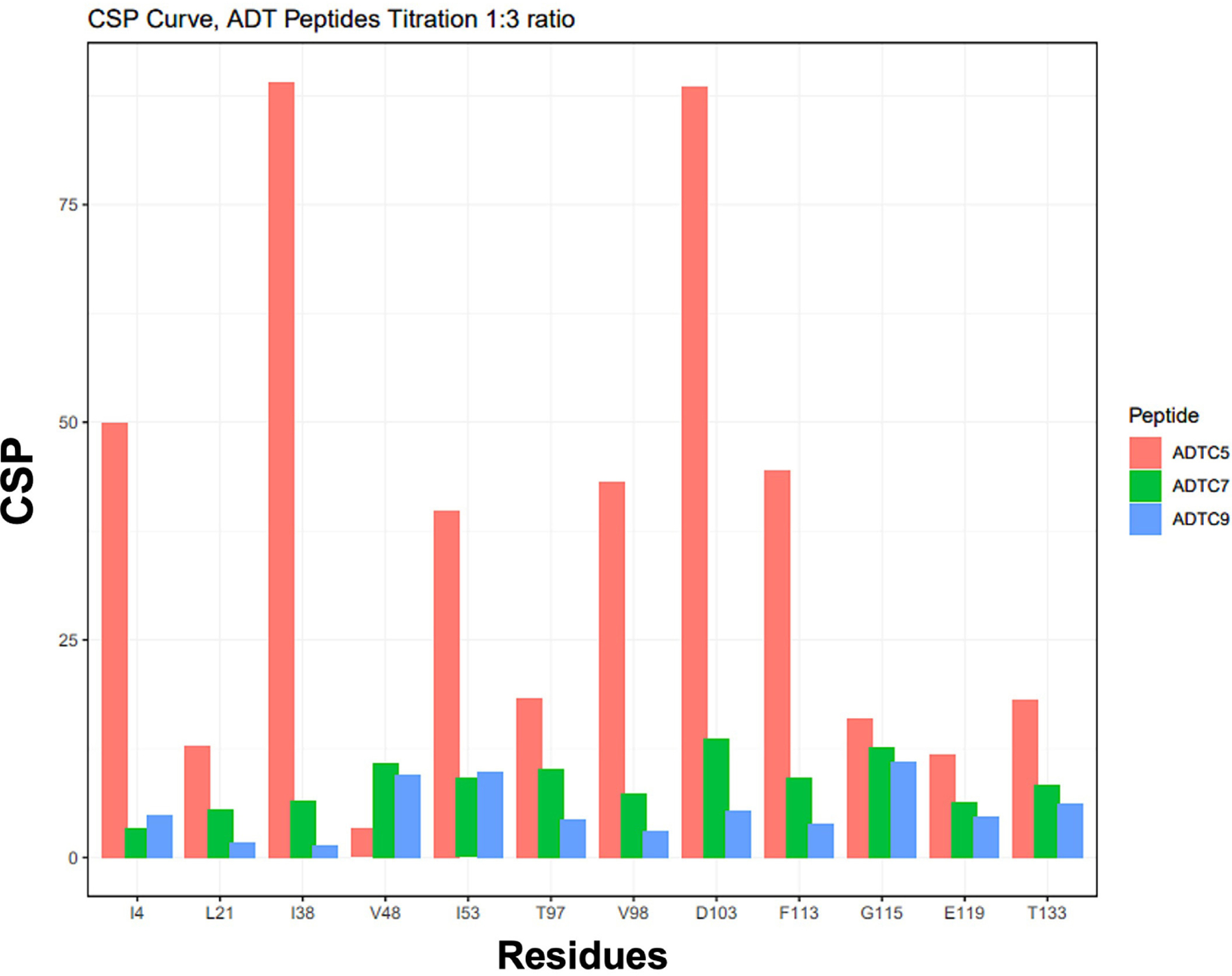
The histogram profile of nuclear magnetic resonance (NMR) chemical shift perturbations (CSPs) on the extracellular-1 (EC1) domain when titrated with Ala-Asp-Thr Cyclic 5 (ADTC5; orange), ADTC7 (green), and ADTC9 (blue) at a 1:3 protein-to-peptide ratio

The titration saturations for both ADTC7 (Figures 1C and 2B) and ADTC9 (Figures 1D and 2C) were at 1:10 and 1:20 protein-to-peptide ratios, respectively. The examples of CSP saturation upon titration of the EC1 domain with ADTC7 and ADTC9 are shown in Figure 2B and C, respectively. Compared to ADTC5, both peptide mutants showed different chemical shift profiles and lower CSP changes for almost all shifting residues (Figure 3). The docking studies indicated that ADTC7 has a similar binding site with ADTC5 at the hydrophobic pocket while ADTC9 binding site is slightly shifted from the binding site of ADTC5. The Kd of ADTC5 is 35 μM while the Kd of ADTC7 is 64 μM using the titration curve of Ile53 residue. Using the titration curve of the Leu21 residue, ADTC9 has a Kd of 181 μM. Therefore, ADTC5 binds better to the EC1 domain compared to ADTC7 and ADTC9. This is also reflected in the CSP comparison among ADTC5, ADTC7, and ADTC9 (Figure 3).

### The change in binding site of ADT peptide upon Val6 mutation

Previous study showed the importance of Val6 residue in ADTC5 to disrupt the BBB intercellular junctions in an in vitro system. To study this effect further, Val6 in ADTC5 was mutated to Glu6 and Tyr6 in ADTC7 and ADTC9, respectively. The EC1 binding characteristics of ADTC7 and ADTC9 were determined by increasing peptide concentrations from 1:0.2 to 1:20 protein-to-peptide ratios. The addition of ADTC7 showed CSPs at the NHs of residues I4, L21, I38, V48, I53, T97, V98, D103, F113, G115, E119, A132, and T133 from the different regions of EC1. The largest changes were observed in V48, I53, D103, and G115 residues. There are some similarities between CSPs in EC1 titrated with ADTC7 and ADTC5; however, a narrower population of CSPs was observed in EC1 titrated with ADTC7 compared to that of ADTC5. Most active residues in EC1 were saturated between 1:10 and 1:15 protein-to-peptide ratios. Upon titration with ADTC9, the largest chemical shift changes in EC1 were at residues L21, V48, I53, and G115 (Figures 1D and 2C). Compared to ADTC5 and ADTC7 peptides, no dramatic CSP change was observed in the D103 residue upon ADTC9 titration, suggesting that the replacement of Val6 to Tyr6 changes the binding location on the EC1 domain.

### Binding clusters of ADT peptides on EC1

The active residues as a binding site on EC1 were determined by the CSPs in amino acids of EC1 upon titration with ADT peptides. From the docking results, all possible docking poses were clustered and analyzed according to their positions. The detailed docking results are summarized in supplemental Table S1.

**Figure 4.**
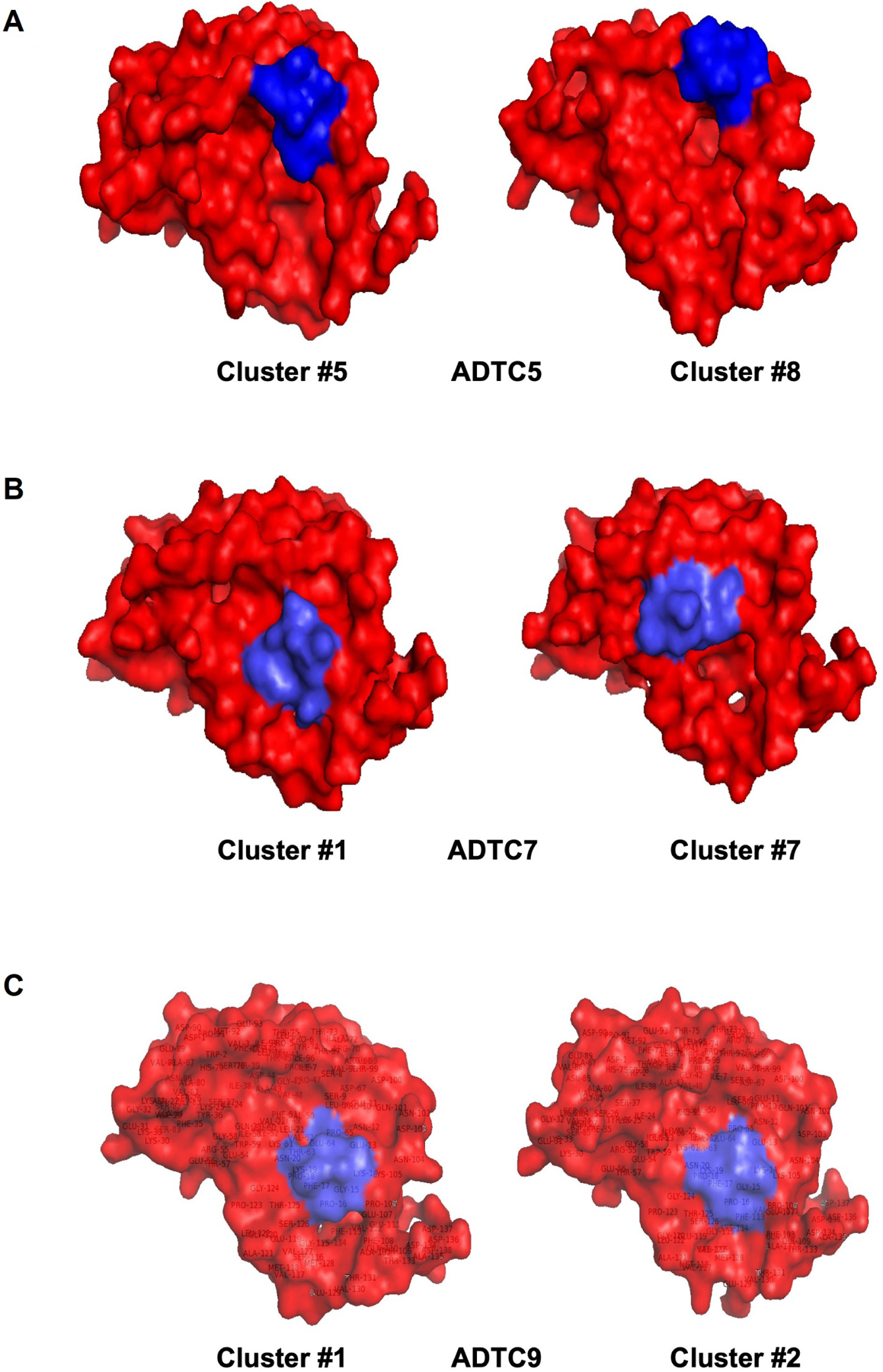
Models of (A) Ala-Asp-Thr Cyclic 5 (ADTC5), (B) ADTC7, and (C) ADTC9 peptides (blue) docked onto the extracellular-1 (EC1) domain (red). (A) Clusters #5 and #8 are used as models for ADTC5-binding to the EC1 domain with a common binding site surrounding residues P6, S8, S9, P10, T97, T99, D100, Q101, D103, and K105; (B) clusters #1 and #7 are representing ADTC7-binding to EC1 and they have a common binding site at E13, K19, N20, L21, K105, P106, G124, S126, and T125 residues; (C) models to represent ADTC9-binding to EC1 are clusters #1 and #2 with a similar binding region around S9, P10, E13, K19, N20, L21, K105, P106, G124, T125, and S126 residues

Clusters #5 and #8 were used as docking models of ADTC5 on EC1 from NMR-constrained docking experiments (Figure 4A). These clusters have the top two highest scores from the HADDOCK program. Cluster #5 of ADTC5 showed EC1 active residues at P6, S8, S9, P10, T97, T99, D100, Q101, D103, and K105. Cluster #8 of ADTC5 docked to EC1 around P6, S8, P10, T99, D103, and K105 residues. Therefore, residues P6, S8, P10, T99, D103, and K105 were the common active site for clusters #5 and #8. All seven clusters have consistent high CSPs on active residues T97, V98, and D103. The least favorable cluster #6 has a binding site located at a different region from the other seven clusters.

From seven model clusters of HADDOCK docking results, ADTC7 was located at the same binding site on EC1 except for one cluster. Clusters #1 and #7 have the two top scores to represent the docking of ADTC7 on the EC1 domain (Figure 4B). The N20, L21, and K105 residues were active residues for clusters #1 and #7; these residues were designated as the binding site of ADTC7 (Table S1). Cluster #1 of ADTC7 has active residues at E13, K19, N20, L21, K105, P106, G124, S126, and T125 as a binding site on EC1. In cluster #7, ADTC7 docked around residues I4, P5, I7, S8, P10, N20, L21, V22, Q23, W59, and K105 on EC1. Both I4 and L21 residues have CSP values upon ADTC7 titration, indicating that they are important for binding to ADTC7. Cluster #6 docked to the tail region of EC1 and some residues on the tail of EC1 showed chemical shift changes upon peptide titration [16].

Six clusters represent docking models of ADTC9 on the EC1 domain and they are located at the same binding site on EC1. Clusters #1 and #2 have the highest scores on HADDOCK results to represent ADTC9 docking on EC1 (Figure 4C). The active residues are S9, P10, E13, K19, N20, L21, K105, P106, T125, G124, and S126 on EC1 for clusters #1 and #2 (Figure 1D). Cluster #1 model specifically interacts with S9, P10, E13, K19, N20, L21, K105, P106, G124, T125, and S126 residues on EC1. Similarly, cluster #2 has active residues S9, P10, E13, P16, N20, L21, W59, T99, K105, F108, P106, G124, T125, and S126 on EC1. The ADTC9 titration produced a high CSP on the L21 that interacts with both clusters #1 and #2 models.

**Figure 5.**
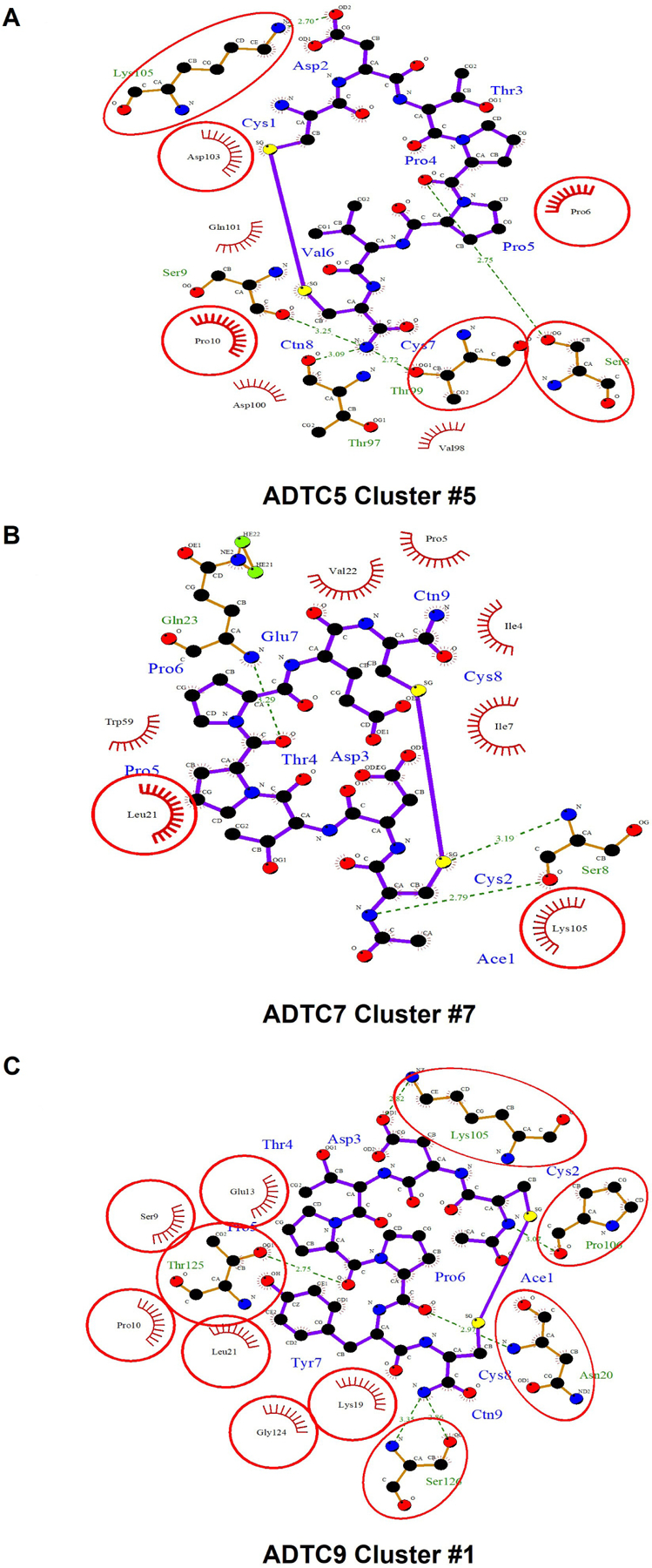
The interactions between amino acids of peptides and the extracellular-1 (EC1) domain represented by one cluster for each peptide: (A) cluster #5 of Ala-Asp-Thr Cyclic 5 (ADTC5), (B) cluster #7 of ADTC7, and (C) cluster #1 of ADTC9

In docking clusters, the 2D interactions of amino acids between peptide and EC1 using representative structures are shown in Figure 5. ADTC5 in cluster #5 showed 5H-bond interactions with S8, S9, T97, T99, and K105 of the EC1 domain (Figure 5A). ADTC7 formed 3H-bonds with S8 and Q23 (Figure 5B). A different set of interactions was found for ADTC9 in which cluster #1 formed H-bonds to N20, K105, P106, T125, and S126 residues on the EC1 domain (Figure 5C). All peptides bind to the hydrophobic pocket of EC1. The protein-ligand interactions for each peptide on its top cluster were summarized in Table S2.

## Discussion

**Figure 6.**
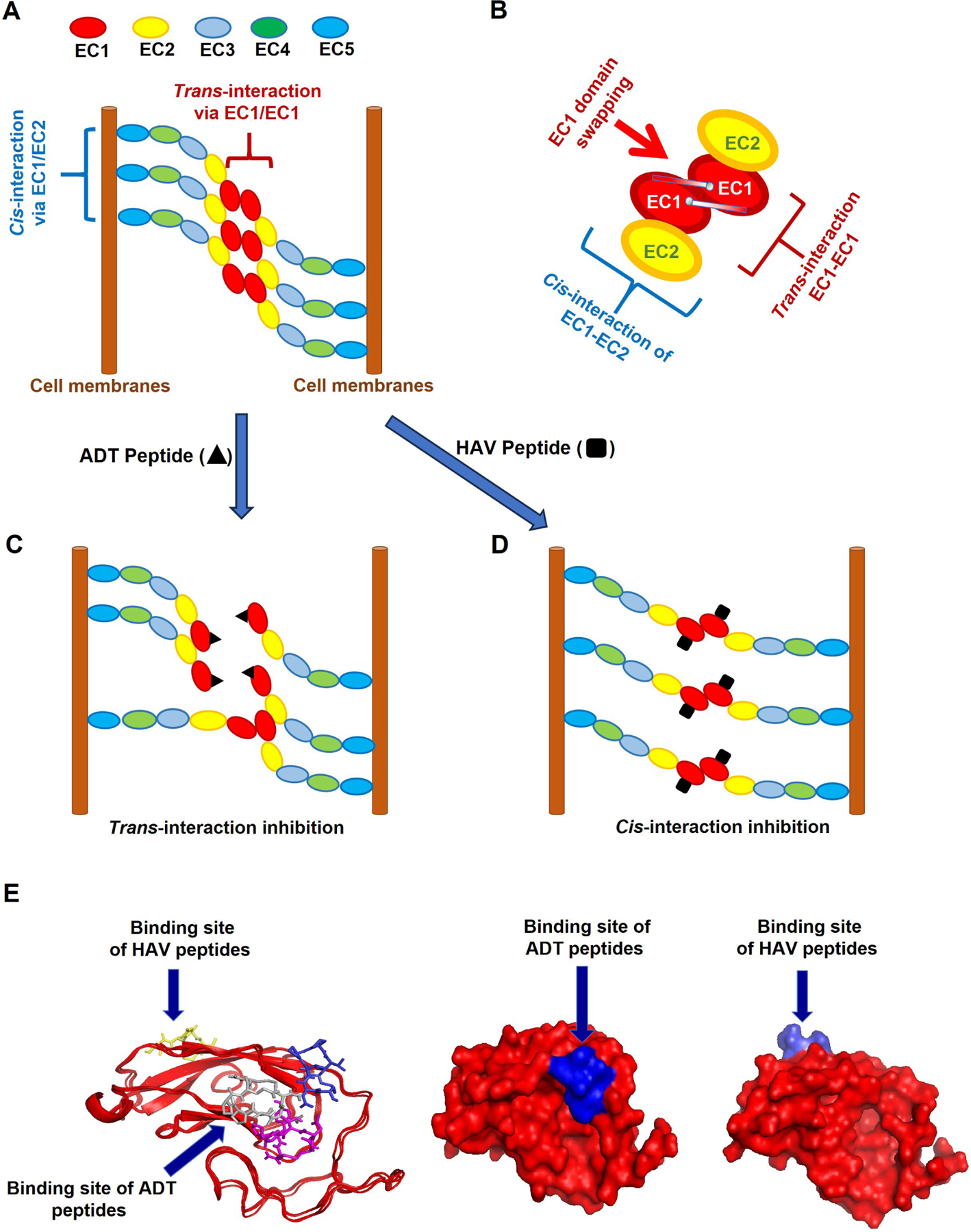
The proposed mechanisms of binding for Ala-Asp-Thr (ADT) and His-Ala-Val (HAV) peptides to extracellular-1 (EC1) domain of cadherin to inhibit cadherin-cadherin interactions. (A) A model to represent cadherin-to-cadherin interactions in the paracellular pathway at the adherens junction. First is the *cis*-interaction of EC1 and EC2 on two cadherin proteins that are protruding from one side of cell membranes. Second is the *trans*-interaction of EC1-EC1 from two cadherins that reside in two opposite membranes; (B) the EC1-EC1 interaction with domain swapping between the N-terminal Trp2 and the hydrophobic pocket of cadherins of the opposing membranes; (C) the proposed binding mechanism of ADT peptides on the hydrophobic region EC1 domain is by hindering *trans*-cadherin interactions to produce high porosity in the paracellular pathway; (D) the proposed binding mechanism of HAV peptides is by preventing *cis*-cadherin interactions to increase porosity in the intercellular junctions; (E) several models show the opposite sites of binding pockets for ADT and HAV peptides on the EC1 surface. These models were determined by nuclear magnetic resonance (NMR) and molecular modeling. These models support the different mechanisms of action for the intercellular junction modulation by ADT and HAV peptides. The binding of HAV peptides to the EC1 domain was represented by cHAVc3 and it was found at the opposite side of ADT cyclic 5 (ADTC5) binding site as shown by Alaofi et al. [16]

ADTC5 modulates the BBB adherens junction in both in vitro and in vivo systems [12, 14, 15, 21]. The EC1 N-terminus has been shown to be the “adhesive arm” of cell-cell adhesion from the crystal structures of C-, E-, and N-cadherins [18, 28]. The adherens junction of biological barriers is composed of *cis*- and *trans*-cadherin-cadherin interactions (Figure 6) [6–8, 17, 19, 20, 29–33]. The *cis*-cadherin interaction is connected by the EC1-EC2 domain-domain interaction of two neighboring cadherins from one side of the membranes (Figure 6A) [6, 7]. The *trans*-cadherin interaction is constructed by EC1-EC1 domain-domain interaction of two cadherins from the opposing cell membranes (Figure 6A) [6, 7]. Furthermore, the interaction involves the domain swapping mechanism (Figure 6B). The domain swapping occurs when the Trp2 residue of the EC1 N-terminal of one cadherin docks to the EC1 hydrophobic pocket of cadherin of the opposing cell membranes and vice versa (Figure 6B) [6, 34–37].

We hypothesized that HAV and ADT peptides prevent cadherin-cadherin interactions by binding to EC1 in the BBB adherens junctions (Figure 6C and D). Cadherin peptides disrupt the integrity of cell-cell adhesion in dynamics and reversible fashion in paracellular pathways of the BBB. Thus, the NMR and molecular docking experiments were carried out to determine the EC1 binding properties of ADT peptides. This study reveals the mechanism of how ADT peptides generate larger junctional pores compared to those in the normal state. ADTC5, ADTC7, and ADTC9 were found to dock at the hydrophobic pocket of the domain swapping region (Figure 6E) [34–37]. Thus, larger than normal pores of the BBB paracellular pathway are created by ADT peptides upon inhibition of the *trans*-cadherin interactions (Figure 6C). The inhibition of *trans*-interactions may still maintain the *cis*-cadherin interactions (Figure 6C). In contrast, cHAVc3 and HAV6 peptides dock on the surface of EC1 that interacts with the EC2 surface from a neighboring cadherin protein to inhibit *cis*-cadherin interaction while maintaining *trans*-interactions (Figure 6D and E). As a result, HAV peptides increase the porosity of the paracellular pathway.

ADT peptides utilize the hydrophobic Val6 and two proline residues for binding to the EC1 hydrophobic pocket that is normally occupied by the N-terminal Trp2 residue during domain swapping [34–37]. Mutations of Val6 in ADTC5 to Glu6 or Tyr6 in ADTC7 or ADTC9, respectively, reduced the binding affinity of ADTC7 or ADTC9 peptide to the EC1 domain, indicating that these peptides bind to the EC1 hydrophobic pocket (Figure 6E) [34–37]. This is consistent with our previous in vitro study where Val6 mutation in ADTC5 reduced the modulatory activity of mutant peptides in MDCK cell monolayers [21]. Furthermore, ADTC7 has an overlap binding pocket with ADTC5 at active residues I4, N20, L21, and K105. ADTC9 has a different binding site from ADTC5; ADTC9 has active residues at S9, E13, N20, L21, K105, P106, T125, and S126 on EC1. In contrast, cHAVc3 peptide as an HAV peptide binds to the opposite side of ADTC5-binding site as previously shown by Alaofi et al. (Figure 6E) [16].

In this study, the EC1 domain consists of a head domain that has seven β-strand along with two short α-helices connected to a 28 amino acid of C-terminal tail, which is derived from the interface region between EC1 and EC2 domains [4, 20, 28, 30]. In MD simulations, the C-terminal tail has been shown to have dynamic mobility that can swing from an extended structure into the head domain to produce a β-sheet interaction as a folded globular structure [16]. It should be noted that the shifted cross peaks upon peptide titrations not only accounted for residues involved in direct binding but also residues participated in conformational change on EC1 away from the binding site. Titration of the EC1 domain with ADTC5 caused the large CSP changes on D103, and F113 residues at the C-terminal tail, indicating that binding of ADTC5 to EC1 may induce conformational change at the C-terminal tail. The D103 residue is in the calcium binding region including PENE, DQND, and LDRE sequences within the EC1-EC2 connecting region [20, 31, 36–39]. Calcium ions at the connecting region between EC domains can convert a globular structure to a rod-like structure of cadherin [36–39]. The large CSP changes were also observed at the I4, I7, and S8 residues at the N-terminus domain upon binding to the ADTC5. This indicates that the binding of ADTC5 can cause conformational change at the N-terminal region or domain swapping region. As noted above, the N-terminal β-sheet has been shown to be dynamic for domain swapping [6, 34–37].

ADTC5 enhanced the delivery of various molecules to the brain using the in-situ rat brain perfusion model [21]. In addition, Gd-DTPA delivery to the brain was enhanced when co-administered with ADTC5 in living mice upon detection with magnetic resonance imaging (MRI) [21]. Next, ADTC5 delivered large molecules into the brain in mice upon intravenous (i.v.) administration and it significantly enhanced brain deposition of peptides, Gd-labeled albumin (Galbumin), and mAb when co-administered with ADTC5 peptide as detected by mass spectrometry, near IR fluorescence (NIRF), and MRI [12, 15]. Brain-derived neurotrophic factor (BDNF) delivery to the brain has been aided by ADTC5 peptide in animal models for AD as well as MS [40, 41]. In an MS mouse model called relapsing-remitting experimental autoimmune encephalomyelitis (RR-EAE), co-administrations of BDNF and ADTC5 during the remission state significantly suppressed the disease relapse in RR-EAE mice compared to those treated with phosphate buffer saline (PBS), ADTC5, and BDNF [40]. Similarly, multiple treatments of APP/PS1 mice as an AD animal model with BDNF + ADTC5 significantly improved the cognitive abilities of mice compared to controls such as PBS- and BDNF-treated mice when evaluated using novel object recognition and Y-maze [41]. In both MS and AD models, treatments with coadministration of BDNF and ADTC5 resulted in the proliferation of NG2 cells that are involved in neuroregeneration.

As BBBMs, the in vivo activities of ADT and HAV peptides to modulate the BBB were transient and reversible [12, 21]. The anatomical structure of the BBB tight junctions did not change after treatment with ADTC5 when determined using transmission electron microscopy (TEM) [21]. Each BBBM has been shown to have an upper limit of the molecular size that it can deliver into the brain. In addition, modulation of the BBB with HAV6 as a BBBM did not induce inflammation and astrogliosis [13]. Different cadherin peptides produced different durations of the BBB opening for different sizes of delivered molecules [12, 21]. ADTC5 created the in vivo paracellular opening for a small molecule such as Gd-DTPA with a duration of less than 4 h [21] while HAV6 peptide generated the BBB opening for the transport of Gd-DTPA in less than 45 min [11]. This indicates that different BBBM peptides generate different durations of BBB modulation. ADTC5 peptide created paracellular porosity for 65 kDa Galbumin in less than 20 min while HAV6 could not deliver Galbumin when there was a delay in the administration of HAV6 and Galbumin [12]. Thus, when compared to Gd-DTPA, the duration window for delivering a large Galbumin was shorter than that of a small Gd-DTPA. ADTC5 peptide had to be administered together with 150 kDa mAb for delivering mAb to the brain while HAV6 could not deliver mAb when administered together [15].

From our previous data, we proposed a working model for BBBM for opening the BBB intercellular junction to improve the delivery of various molecules. In this case, we propose that BBBM peptides generate large, medium, and small pores with different durations of pore openings (different half-lives). The generated large pores collapse quickly to medium and small pores; this is supported by the data from the brain delivery of albumin (65 kDa) and IgG mAb (150 kDa) using ADTC5. Thus, the duration of BBB penetration of medium and small size molecules will be longer than the large molecules. Similarly, the medium pores collapse into small pores and finally, the small pores disappear into normal pores of the intercellular junction. This suggests that the pore opening was time- and size-dependent.

Several other methods have been investigated to improve the brain delivery of drugs and diagnostic agents [2, 42]. Similar to our method, the osmotic (OSM) BBB disruption method has been successfully developed and used by Neuwelt et al. [43–45] to deliver anticancer agents to treat brain tumors in patients. This method uses a hypertonic mannitol solution to drive fluid out of the BBB endothelial cells resulting in cell shrinkage to increase paracellular porosity of the intercellular junctions to allow penetration of drugs into the brain. Unfortunately, long-term disruption of BBB tight junctions may induce astrogliosis or brain inflammation [46].

More recently, focused ultrasound (FUS) has also been used to locally disrupt tight junctions, and in combination with microbubbles, the method has been effectively used to deliver drugs to the brain [47, 48]. The disadvantages of both OSM and FUS disruption of the BBB reside in the inability to control the duration and magnitude of effect resulting in relatively large-scale BBB openings for long (8 h or more) periods of time leading to astrogliosis or brain inflammation [46]. Another method to increase the BBB porosity is the bradykinin receptor agonist, Cereport^™^ (RMP-7) [49]. This method had been used in several animal models to improve drug delivery into the brain; however, this method had difficulty modulating the BBB consistently. Finally, a claudin peptide (C1C2) was found to increase the porosity of the BBB tight junctions and improve brain delivery of an opioid peptide and tetrodotoxin [50]. For drugs that are substrates for the efflux pump, their co-administration with an efflux pump inhibitor (e.g., verapamil and cyclosporine A) can enhance transcellular drug penetration through the BBB [51].

The receptor-mediated endocytosis process has been extensively explored to deliver drugs across the BBB. MAbs or antigen-binding fragments (Fabs) to the transferrin receptor (TfR) have been used successfully to carry drugs across the BBB [2]. Conjugation of TfR mAbs with enzymes (e.g., β galactosidase-1, iduronate-2-sulfatase) has been investigated for their use in enzyme replacement therapy in lysosomal disorders (e.g., Niemann-Pick disease) [52]. The Angiopep-2 peptide conjugated with other peptides such as β-secretase inhibitor (SI) and paclitaxel inhibited the production of amyloid-beta (Aβ) in neuronal cells and killed brain tumor cells, respectively [53, 54]. Cell-penetrating peptides (CPPs) have been used for brain delivery of drugs and β-galactosidase [55].

Another method to deliver drugs to the brain is via the intranasal delivery route; this method bypasses the BBB. In this case, the delivered drug crosses the nasal epithelial layer followed by penetrating through the cribriform plate to diffuse into the olfactory bulb and the trigeminal nerve. Insulin and oxytocin have been delivered via intranasal and they have been evaluated in clinical trials for treating autism spectrum disorder (ASD) and AD [56]. It has been shown that nasal delivery of peptide was more effective than that of i.v. and intraperitoneal (i.p.) delivery; this is because the peptide did not enter the systemic circulation and was degraded by enzymes in the plasma [57]. It should be noted that a fraction of peptide delivered intranasally is distributed to the systemic circulation; thus, this portion needs to cross the BBB to enter the brain. Because delivered peptides need to penetrate the nasal epithelial layer, some peptide formulations contain tight junction modulators (i.e., carnitines) and CPP as well as delivered in the presence of ultrasound [57]. Some intranasal delivery methods are still in clinical trials while some have been approved to treat patients with CNS disorders.

In conclusion, ADTC5 and its derivatives dock to the hydrophobic pocket of the domain swapping mechanism for the EC1-EC1 domain interactions. Therefore, ADT peptides have a mechanism of action to modulate the BBB through the prevention of *trans*-cadherin interactions to cause molecules to percolate across the BBB into the brain in time dependent manner. In the future, this bound structure of ADTC5 will be used to design derivatives that have enhanced binding properties to the EC1 domain that can be used to improve the modulation of the BBB.

## Supplementary Material

Supplementary Material

## Figures and Tables

**Table 1.** Synthetic ADT and HAV peptides

Peptides	Sequences	Molecular weight (MW, atomic mass unit)
ADTC5	Cyclo(1,7)Ac-CDTPPVC-NH_2_	744
ADTC7	Cyclo(1,7)Ac-CDTPPEC-NH_2_	803
ADTC9	Cyclo(1,7)Ac-CDTPPYC-NH_2_	837
cHAVc3	Cyclo(1,6)Ac-CSHAVC-NH_2_	657
HAV6	Ac-SHAVSS-NH_2_	628

ADT: Ala-Asp-Thr; HAV: His-Ala-Val; ADTC5: ADT cyclic 5; cHAVc3: cyclic HAV-Cys 3

## Data Availability

The data used for this study are included in the manuscript and the supplementary files.

## Abbreviations

2D: two dimensional
AD: Alzheimer’s disease
ADT: Ala-Asp-Thr
ADTC5: Ala-Asp-Thr Cyclic 5
BBB: blood-brain barrier
BBBM: blood-brain barrier modulator
BDNF: brain-derived neurotrophic factor
cHAVc3: cyclic His-Ala-Val-Cys 3
CSP: chemical shift perturbation
EC1: extracellular-1
Galbumin: gadolinium-labeled albumin
Gd-DTPA: gadolinium-diethylenetriaminepentacetate
HAV: His-Ala-Val
HPLC: high performance liquid chromatography
HSQC: heteronuclear single quantum coherence
mAb: monoclonal antibody
MD: molecular dynamics
MS: multiple sclerosis
NMR: nuclear magnetic resonance

## References

[1] Laksitorini M, Prasasty VD, Kiptoo PK, Siahaan TJ. Pathways and progress in improving drug delivery through the intestinal mucosa and blood-brain barriers. Ther Deliv. 2014;5:1143–63. 25418271 10.4155/tde.14.67 PMC4445828

[2] Pardridge WM. A Historical Review of Brain Drug Delivery. Pharmaceutics. 2022;14:1283. 35745855 10.3390/pharmaceutics14061283 PMC9229021

[3] Stamatovic SM, Johnson AM, Keep RF, Andjelkovic AV. Junctional proteins of the blood-brain barrier: New insights into function and dysfunction. Tissue Barriers. 2016;4:e1154641. 27141427 10.1080/21688370.2016.1154641 PMC4836471

[4] Hartsock A, Nelson WJ. Adherens and tight junctions: structure, function and connections to the actin cytoskeleton. Biochim Biophys Acta. 2008;1778:660–9. 17854762 10.1016/j.bbamem.2007.07.012 PMC2682436

[5] Adson A, Raub TJ, Burton PS, Barsuhn CL, Hilgers AR, Audus KL, Quantitative approaches to delineate paracellular diffusion in cultured epithelial cell monolayers. J Pharm Sci. 1994;83:1529–36. 7891269 10.1002/jps.2600831103

[6] Boggon TJ, Murray J, Chappuis-Flament S, Wong E, Gumbiner BM, Shapiro L. C-cadherin ectodomain structure and implications for cell adhesion mechanisms. Science. 2002;296:1308–13. 11964443 10.1126/science.1071559

[7] Zheng K, Trivedi M, Siahaan TJ. Structure and function of the intercellular junctions: barrier of paracellular drug delivery. Curr Pharm Des. 2006;12:2813–24. 16918412 10.2174/138161206777947722

[8] Nagar B, Overduin M, Ikura M, Rini JM. Structural basis of calcium-induced E-cadherin rigidification and dimerization. Nature. 1996;380:360–4. 8598933 10.1038/380360a0

[9] Sinaga E, Jois SD, Avery M, Makagiansar IT, Tambunan US, Audus KL, Increasing paracellular porosity by E-cadherin peptides: discovery of bulge and groove regions in the EC1-domain of E-cadherin. Pharm Res. 2002;19:1170–9. 12240943 10.1023/a:1019850226631

[10] Kiptoo P, Sinaga E, Calcagno AM, Zhao H, Kobayashi N, Tambunan US, Enhancement of drug absorption through the blood-brain barrier and inhibition of intercellular tight junction resealing by E-cadherin peptides. Mol Pharm. 2011;8:239–49. 21128658 10.1021/mp100293m PMC3078649

[11] On NH, Kiptoo P, Siahaan TJ, Miller DW. Modulation of blood-brain barrier permeability in mice using synthetic E-cadherin peptide. Mol Pharm. 2014;11:974–81. 24495091 10.1021/mp400624v PMC3993937

[12] Ulapane KR, On N, Kiptoo P, Williams TD, Miller DW, Siahaan TJ. Improving Brain Delivery of Biomolecules via BBB Modulation in Mouse and Rat: Detection using MRI, NIRF, and Mass Spectrometry. Nanotheranostics. 2017;1:217–31. 28890866 10.7150/ntno.19158 PMC5588751

[13] Sajesh BV, On NH, Omar R, Alrushaid S, Kopec BM, Wang WG, Validation of Cadherin HAV6 Peptide in the Transient Modulation of the Blood-Brain Barrier for the Treatment of Brain Tumors. Pharmaceutics. 2019;11:481. 31533285 10.3390/pharmaceutics11090481 PMC6781504

[14] Ulapane KR, Kopec BM, Siahaan TJ. Improving In Vivo Brain Delivery of Monoclonal Antibody Using Novel Cyclic Peptides. Pharmaceutics. 2019;11:568. 31683745 10.3390/pharmaceutics11110568 PMC6920923

[15] Ulapane KR, Kopec BM, Siahaan TJ. In Vivo Brain Delivery and Brain Deposition of Proteins with Various Sizes. Mol Pharm. 2019;16:4878–89. 31664837 10.1021/acs.molpharmaceut.9b00763 PMC8554818

[16] Alaofi A, Farokhi E, Prasasty VD, Anbanandam A, Kuczera K, Siahaan TJ. Probing the interaction between cHAVc3 peptide and the EC1 domain of E-cadherin using NMR and molecular dynamics simulations. J Biomol Struct Dyn. 2017;35:92–104. 26728967 10.1080/07391102.2015.1133321 PMC5061580

[17] Takeda H *cis*-Dimer formation of E-cadherin is independent of cell-cell adhesion assembly in vivo. Biochem Biophys Res Commun. 2004;316:822–6. 15033474 10.1016/j.bbrc.2004.02.123

[18] Chappuis-Flament S, Wong E, Hicks LD, Kay CM, Gumbiner BM. Multiple cadherin extracellular repeats mediate homophilic binding and adhesion. J Cell Biol. 2001;154:231–43. 11449003 10.1083/jcb.200103143 PMC2196848

[19] Takeda H, Shimoyama Y, Nagafuchi A, Hirohashi S. E-cadherin functions as a *cis*-dimer at the cell-cell adhesive interface *in vivo*. Nat Struct Biol. 1999;6:310–2. 10201395 10.1038/7542

[20] Pertz O, Bozic D, Koch AW, Fauser C, Brancaccio A, Engel J. A new crystal structure, Ca^2+^ dependence and mutational analysis reveal molecular details of E-cadherin homoassociation. EMBO J. 1999;18: 1738–47. 10202138 10.1093/emboj/18.7.1738 PMC1171260

[21] Laksitorini MD, Kiptoo PK, On NH, Thliveris JA, Miller DW, Siahaan TJ. Modulation of intercellular junctions by cyclic-ADT peptides as a method to reversibly increase blood-brain barrier permeability. J Pharm Sci. 2015;104:1065–75. 25640479 10.1002/jps.24309 PMC4442687

[22] Prasasty VD, Krause ME, Tambunan US, Anbanandam A, Laurence JS, Siahaan TJ. ^1^H, ^13^C and ^15^N backbone assignment of the EC-1 domain of human E-cadherin. Biomol NMR Assign. 2015;9:31–5. 24510398 10.1007/s12104-013-9539-6 PMC4133310

[23] Lee W, Tonelli M, Markley JL. NMRFAM-SPARKY: enhanced software for biomolecular NMR spectroscopy. Bioinformatics. 2015;31:1325–7. 25505092 10.1093/bioinformatics/btu830 PMC4393527

[24] Dominguez C, Boelens R, Bonvin AM. HADDOCK: a protein-protein docking approach based on biochemical or biophysical information. J Am Chem Soc. 2003;125:1731–7. 12580598 10.1021/ja026939x

[25] Bonvinlab. HADDOCK. Version 2.4 [Software]. [cited 2020 Jun]. Available from: https://wenmr.science.uu.nl/haddock2.4/

[26] Laskowski RA, Swindells MB. LigPlot+: multiple ligand-protein interaction diagrams for drug discovery. J Chem Inf Model. 2011;51:2778–86. 21919503 10.1021/ci200227u

[27] Skinner AL, Laurence JS. Probing residue-specific interactions in the stabilization of proteins using high-resolution NMR: a study of disulfide bond compensation. J Pharm Sci. 2010;99:2643–54. 20187138 10.1002/jps.22055 PMC3683975

[28] Parisini E, Higgins JM, Liu JH, Brenner MB, Wang JH. The crystal structure of human E-cadherin domains 1 and 2, and comparison with other cadherins in the context of adhesion mechanism. J Mol Biol. 2007;373:401–11. 17850815 10.1016/j.jmb.2007.08.011 PMC2094043

[29] Angst BD, Marcozzi C, Magee AI. The cadherin superfamily: diversity in form and function. J Cell Sci. 2001;114:629–41. 11171368 10.1242/jcs.114.4.629

[30] van Roy F, Berx G. The cell-cell adhesion molecule E-cadherin. Cell Mol Life Sci. 2008;65:3756–88. 18726070 10.1007/s00018-008-8281-1 PMC11131785

[31] Koch AW, Manzur KL, Shan W. Structure-based models of cadherin-mediated cell adhesion: the evolution continues. Cell Mol Life Sci. 2004;61:1884–95. 15289931 10.1007/s00018-004-4006-2 PMC11138478

[32] Koch AW, Pokutta S, Lustig A, Engel J. Calcium binding and homoassociation of E-cadherin domains. Biochemistry. 1997;36:7697–705. 9201910 10.1021/bi9705624

[33] Troyanovsky RB, Sokolov E, Troyanovsky SM. Adhesive and lateral E-cadherin dimers are mediated by the same interface. Mol Cell Biol. 2003;23:7965–72. 14585958 10.1128/MCB.23.22.7965-7972.2003 PMC262383

[34] Patel SD, Ciatto C, Chen CP, Bahna F, Rajebhosale M, Arkus N, Type II cadherin ectodomain structures: implications for classical cadherin specificity. Cell. 2006;124:1255–68. 16564015 10.1016/j.cell.2005.12.046

[35] Miloushev VZ, Bahna F, Ciatto C, Ahlsen G, Honig B, Shapiro L, Dynamic properties of a type II cadherin adhesive domain: implications for the mechanism of strand-swapping of classical cadherins. Structure. 2008;16:1195–205. 18682221 10.1016/j.str.2008.05.009 PMC2561228

[36] Vunnam N, Pedigo S. Calcium-induced strain in the monomer promotes dimerization in neural cadherin. Biochemistry. 2011;50:8437–44. 21870846 10.1021/bi200902s

[37] Davila S, Liu P, Smith A, Marshall AG, Pedigo S. Spontaneous Calcium-Independent Dimerization of the Isolated First Domain of Neural Cadherin. Biochemistry. 2018;57:6404–15. 30387993 10.1021/acs.biochem.8b00733

[38] Overduin M, Harvey TS, Bagby S, Tong KI, Yau P, Takeichi M, Solution structure of the epithelial cadherin domain responsible for selective cell adhesion. Science. 1995;267:386–9. 7824937 10.1126/science.7824937

[39] Kim SA, Tai CY, Mok LP, Mosser EA, Schuman EM. Calcium-dependent dynamics of cadherin interactions at cell-cell junctions. Proc Natl Acad Sci U S A. 2011;108:9857–62. 21613566 10.1073/pnas.1019003108 PMC3116393

[40] Kopec BM, Kiptoo P, Zhao L, Rosa-Molinar E, Siahaan TJ. Noninvasive Brain Delivery and Efficacy of BDNF to Stimulate Neuroregeneration and Suppression of Disease Relapse in EAE Mice. Mol Pharm. 2020;17:404–16. 31846344 10.1021/acs.molpharmaceut.9b00644 PMC10088282

[41] Kopec BM, Zhao L, Rosa-Molinar E, Siahaan TJ. Non-invasive Brain Delivery and Efficacy of BDNF in APP/PS1 Transgenic Mice as a Model of Alzheimer’s Disease. Med Res Arch. 2020;8:2043. 32551362 10.18103/mra.v8i2.2043 PMC7302105

[42] Elballa W, Schwinghamer K, Ebert E, Siahaan TJ. Peptides and Their Delivery to the Brain. In: Jois SD, editor. Peptide Therapeutics: Fundamentals of Design, Development, and Delivery. Cham: Springer International Publishing; 2022. pp. 237–66.

[43] Doolittle ND, Muldoon LL, Culp AY, Neuwelt EA. Delivery of chemotherapeutics across the blood-brain barrier: challenges and advances. Adv Pharmacol. 2014;71:203–43. 25307218 10.1016/bs.apha.2014.06.002 PMC5505259

[44] Smith QR. Quantitation of Blood-Brain Barrier Permeability. In: Neuwelt EA, editor. Implications of the Blood-Brain Barrier and Its Manipulation: Volume 1 Basic Science Aspects. Boston, MA: Springer US; 1989. pp. 85–118.

[45] Neuwelt EA, Barnett PA, McCormick CI, Frenkel EP, Minna JD. Osmotic blood-brain barrier modification: monoclonal antibody, albumin, and methotrexate delivery to cerebrospinal fluid and brain. Neurosurgery. 1985;17:419–23. 3930991 10.1227/00006123-198509000-00004

[46] Neuwelt EA, Specht HD, Barnett PA, Dahlborg SA, Miley A, Larson SM, Increased delivery of tumor-specific monoclonal antibodies to brain after osmotic blood-brain barrier modification in patients with melanoma metastatic to the central nervous system. Neurosurgery. 1987;20:885–95. 3112602 10.1227/00006123-198706000-00011

[47] Fishman PS, Fischell JM. Focused Ultrasound Mediated Opening of the Blood-Brain Barrier for Neurodegenerative Diseases. Front Neurol. 2021;12:749047. 34803886 10.3389/fneur.2021.749047 PMC8599441

[48] Iyer J, Akkad A, Tang N, Berens M, Zenhausern F, Gu J. EXTH-17. A FOCUSED ULTRASOUND BLOOD BRAIN BARRIER DISRUPTION MODEL TO TEST THE INFLUENCE OF TIGHT JUNCTION GENES TO TREAT BRAIN TUMORS. Neuro-Oncol. 2021;23:vi167.

[49] Borlongan CV, Emerich DF. Facilitation of drug entry into the CNS via transient permeation of blood brain barrier: laboratory and preliminary clinical evidence from bradykinin receptor agonist, Cereport. Brain Res Bull. 2003;60:297–306. 12754091 10.1016/s0361-9230(03)00043-1

[50] Zwanziger D, Hackel D, Staat C, Böcker A, Brack A, Beyermann M, A peptidomimetic tight junction modulator to improve regional analgesia. Mol Pharm. 2012;9:1785–94. 22524793 10.1021/mp3000937

[51] Ansbro MR, Shukla S, Ambudkar SV, Yuspa SH, Li L. Screening compounds with a novel high-throughput ABCB1-mediated efflux assay identifies drugs with known therapeutic targets at risk for multidrug resistance interference. PLoS One. 2013;8:e60334. 23593196 10.1371/journal.pone.0060334 PMC3622673

[52] Zhou Z, Austin GL, Shaffer R, Armstrong DD, Gentry MS. Antibody-Mediated Enzyme Therapeutics and Applications in Glycogen Storage Diseases. Trends Mol Med. 2019;25:1094–109. 31522955 10.1016/j.molmed.2019.08.005 PMC6889062

[53] Li Y, Zheng X, Gong M, Zhang J. Delivery of a peptide-drug conjugate targeting the blood brain barrier improved the efficacy of paclitaxel against glioma. Oncotarget. 2016;7:79401–7. 27765902 10.18632/oncotarget.12708 PMC5346723

[54] Kim JA, Casalini T, Brambilla D, Leroux JC. Presumed LRP1-targeting transport peptide delivers β-secretase inhibitor to neurons in vitro with limited efficiency. Sci Rep. 2016;6:34297. 27682851 10.1038/srep34297 PMC5041153

[55] Oller-Salvia B, Sánchez-Navarro M, Giralt E, Teixidó M. Blood-brain barrier shuttle peptides: an emerging paradigm for brain delivery. Chem Soc Rev. 2016;45:4690–707. 27188322 10.1039/c6cs00076b

[56] Tanaka A, Furubayashi T, Arai M, Inoue D, Kimura S, Kiriyama A, Delivery of Oxytocin to the Brain for the Treatment of Autism Spectrum Disorder by Nasal Application. Mol Pharm. 2018;15: 1105–11. 29338251 10.1021/acs.molpharmaceut.7b00991

[57] Samaridou E, Alonso MJ. Nose-to-brain peptide delivery – The potential of nanotechnology. Bioorg Med Chem. 2018;26:2888–905. 29170026 10.1016/j.bmc.2017.11.001

