# Supplementary material for: Mechanism of the blood-brain barrier modulation by cadherin peptides
Source: Explor Drug Sci. Author manuscript; Available in PMC 2024 Aug 8. (PMC11309765; doi:10.37349/eds.2024.00049)
Supplement: Supplementary Material [file NIHMS2011639-supplement-Supplementary_Material.docx]

**Supplementary Material**

**Mechanism of the blood-brain barrier modulation by cadherin peptides**

**Table S1. Interacting EC1 residues for each HADDOCK cluster per peptide**

| **ADT Peptide** | **Interacting EC1 Domain Residues** |
| --- | --- |
| **ADTC5** | *Cluster#1* S8, S9, P10, T99, D100, Q101, N102, D103, K105,  *Cluster#2* P6, R68, T73, L95, T97, V98, T99, D100  *Cluster#3* P5, P6, I7, S8, P10, L21, T99 K105  *Cluster#4* I7, S8, S9, P10 T99, D100, Q101, N102, D103, N104, K105  *Cluster#5* P6, S8, S9, P10, T97, T99, D100, Q101, D103, K105  *Cluster#6** Q23, K25, E56, G58, S26, N27, Y36, I24, D29, K30  *Cluster#7* W2, V3, P5, P6, I7, S8, L21, V22, Q23, W59  *Cluster#8* P6, S8, P10, T99D103, K105 |
| **ADTC7** | *Cluster#1* E13, K19, N20, L21, K105, P106, G124, S126, T125  *Cluster#2* I7, P10, E13, K19, N20, L21, K105, P106, T125, S126  *Cluster#3*  G15, P18, K19, N20, L21, P106, Glu107, F108, T125, S126, A132  *Cluster#4* I4, P5, P6, I7, S8 L21K105, V122  *Cluster#5* I7, S8, E13, K19, N20, L21, W59 K105, P106, T125  *Cluster#6** T109, F113, K114, G115, S116, M128, V130  *Cluster#7* I4, P5, I7, S8, P10, N20, L21, V22, Q23, K105, W59 |
| **ADTC9** | *Cluster#1* S9, P10, E13, K19, N20, L21, K105, P106, T125, G124, S126  *Cluster#2* S9, P10, E13, P16, N20, L21, W59, T99, K105, F108, P106, T125, G124, S126  *Cluster#3* E13, P16, K19, N20, L21, K105, P106, T125, G124, Ser126  *Cluster#4* I4, P5, P6, I7, S8, P10, N20, L21, V22, W59, K105  *Cluster#5* P16, K19, N20, K105, P106, E107, F108, T125, S126, A132  *Cluster#6* P5, P6, I7, S8, P10, E13, N20, L21, D103, K105 |

* Represent models that were docked differently from the other models for ADTc7 or ADTc5 peptides.

**Table S2. Protein-Ligand Interactions**

| **ADT peptides** | **Cluster** | **Hydrogen bond** | | **Non-bonded Interaction** |
| --- | --- | --- | --- | --- |
|  |  | **EC1 residue** | **Peptide residue** |  |
| ADTC5 | #5 | Lys105 | Asp2 sidechain | Asp103 |
|  |  | Ser8 | Pro5 carbonyl backbone | Pro6 |
|  |  | Thr99 | Amide nitrogen- C terminus | Gln101 |
|  |  | Thr97 | Amide nitrogen- C terminus | Pro10 |
|  |  | Ser9 | Amide nitrogen- C terminus | Asp100 |
|  |  |  |  | Val98 |
| ADTC7 | #7 | Gln23 | Pro5 carbonyl backbone | Pro5 |
|  |  | Ser8 | Cys8 S sidechain | Val22 |
|  |  |  | Cys8 N terminus | Ile4 |
|  |  |  |  | Ile7 |
|  |  |  |  | Lys105 |
|  |  |  |  | Pro10 |
|  |  |  |  | Leu21 |
|  |  |  |  | Trp59 |
| ADTC9 | #1 | Lys105 | Asp3 sidechain | Glu13 |
|  |  | Pro106 | Cys1 N terminus | Ser9 |
|  |  | Asn20 | Pro6 carbonyl backbone | Pro10 |
|  |  | Ser126 | Amide nitrogen- C terminus (2x) | Leu21 |
|  |  | Thr125 | Pro5 carbonyl backbone | Gly124 |
|  |  |  |  | Lys19 |
